# Polygenic risk and air pollution trends in relation to type 2 diabetes: evidence from the Taiwan Biobank

**DOI:** 10.1186/s13098-026-02088-1

**Published:** 2026-01-20

**Authors:** Osama Aziz, Bing-Fang Hwang, Ai-Ru Hsieh, Chau-Ren Jung

**Affiliations:** 1 Department of Public Health, College of Public Health, China Medical University, No. 100, Sec. 1, Jingmao Rd. Beitun Dist., Taichung, 406040 Taiwan; 2 Department of Occupational Safety and Health, College of Public Health, China Medical University, Taichung, Taiwan; 3 Department of Statistics, National Taipei University, No.151, University Rd., San Shia District, New Taipei, 23741 Taiwan; 4 Japan Environment and Children’s Study Programme Office, National Institute for Environmental Studies, Tsukuba, Japan

**Keywords:** Air pollution, Gene-Environment interaction, Polygenic risk scores, PM_2.5_, Taiwan biobank, Type 2 diabetes mellitus

## Abstract

**Background:**

Both genetic predisposition and air pollution may lead to type 2 diabetes mellitus (T2D). However, the synergistic effects of gene by environment interactions between air pollution exposure and genetic susceptibility to T2D remain underexplored in Asian populations. This study assessed the association between air pollutants, including fine particulate matter (PM_2.5_), nitrogen dioxide (NO_2_), sulfur dioxide (SO_2_), and ozone (O_3_), and T2D, while considering polygenic risk scores (PRS).

**Methods:**

Data were obtained from 104,554 participants in the Taiwan Biobank. Air pollutant concentrations were estimated using satellite-based models, and long-term trends were represented by slopes derived from linear regression models. The PRS for T2D was constructed from East Asian-specific genome-wide association study summary statistics (AGEN consortium) using the clumping and thresholding method. Logistic regression models were applied to examine associations of T2D with air pollution and PRS, expressed as odds ratios (ORs) and 95% confidence intervals (CIs). Additive interaction was evaluated using the relative excess risk due to interaction (RERI), and multiplicative interaction was tested via cross-product terms in logistic models.

**Results:**

Every 1 µg/m^3^ per year increase in PM_2.5_ concentrations was significantly associated with increased T2D risk (OR: 1.036, 95% CI: 1.003–1.071). A positive exposure-response relationship between PRS and T2D was observed, with individuals in the highest PRS quartile showing significantly higher risk (OR: 1.385, 95% CI: 1.279,1.499). The association between PM_2.5_ slope and T2D was slightly stronger among those with the highest genetic risk; however, the additive interaction was weak and borderline significant (RERI: 0.144, 95% CI: 0.008–0.319).

**Conclusions:**

Both worsening PM_2.5_ exposure and PRS were associated with T2D. The observed PM_2.5_ and PRS interaction was weak and should be interpreted cautiously. Our findings highlight the importance of improving air quality and adopting personalized prevention strategies for individuals with high genetic risk.

**Supplementary Information:**

The online version contains supplementary material available at 10.1186/s13098-026-02088-1.

## Background

Diabetes mellitus (DM) is a growing global public health crisis, recognized as a major threat by the World Health Organization (WHO) [1]. The prevalence of DM among adults aged 20–79 years reached approximately 415 million in 2015 and is projected to increase to 642 million cases by 2040 [2], presenting an immense challenge to healthcare systems worldwide, with type 2 diabetes mellitus (T2D) accounting for the vast majority of cases (approximately 99.4%) [2]. In Taiwan, the burden of DM mirrors this global trend, remaining a leading cause of death for many years [3], with the overall prevalence of DM rising sharply between 2005 and 2014, increasing by 59% in men and 54% in women [3]. Furthermore, over half (50.3%) of affected individuals are aged 65 years or older, with prevalence historically elevated in elderly women [3]. Given the escalating incidence and substantial social burden of T2D, it is critically important to identify modifiable risk factors that could contribute to effective prevention and management strategies. Long-term exposure to ambient air pollution is now recognized as a significant, modifiable environmental risk factor for T2D [4, 5]. In particular, particulate matter with an aerodynamic diameter less than 2.5 μm (PM_2.5_) has been consistently associated with an increased risk [4]. A landmark study demonstrated that a 10 µg/m^3^ increase in long-term PM_2.5_ exposure was associated with a 26% higher risk of T2D (HR:1.26, 95% CI: 1.22 to 1.31) after adjusting for confounders [5]. Furthermore, a comprehensive meta-analysis revealed a 28% augmented risk correlated with PM_2.5_ exposure, with analogous relationships observed for nitrogen dioxide (NO_2_) [6, 7]. Epidemiological studies consistently show that T2D disproportionately affects vulnerable populations in populous urban areas and low-income communities [8, 9]. This disparity highlights the potential for synergistic interactions between genetic vulnerabilities and environmental exposures [8, 9]. Indeed, recent evidence confirms that the combined effect of elevated PM_2.5_ exposure and high genetic risk scores significantly increases T2D risk (HRs:1.28–1.35), with a notable relative excess risk due to interaction (RERI) of 0.37 for high-risk individuals [10]. This demonstrates that the interaction of genetic and environmental factors significantly link T2D development [10]. A polygenic risk score (PRS) quantifies an individual’s genetic risk for disease by combining the cumulative effects of numerous single nucleotide polymorphisms (SNPs), each weighted by effect sizes from genome-wide association studies (GWAS) [11, 12]. PRS is a valuable tool for early disease prediction [13] and clinical integration [14, 15], but its predictive effectiveness varies significantly by ancestry, underscoring the critical need for population-specific approaches [13]. For instance, studies have shown that South Asian-derived PRS models substantially outperform European-derived models in non-European cohorts, and combining PRS with clinical risk scores significantly improves overall predictive accuracy (area under the curve [AUC] from 0.72 − 0.74 to 0.75 − 0.79) [16]. These findings indicate the necessity of incorporating genetic factors to enhance T2D risk prediction in underrepresented cohorts. While the harmful effects of air pollution on chronic conditions are well-documented, limited research has examined the interaction between PRS and air pollution exposure in influencing T2D risk, especially in Asian populations [10, 17]. This knowledge gap highlights the urgent need for comprehensive studies that integrate these distinct risk factors to elucidate their combined impact on T2D within ancestrally relevant cohorts.

Understanding the combined associations of genetic predisposition and air pollution exposure is vital for developing precision prevention strategies for T2D. This study leveraged the Taiwan Biobank (TWB), a comprehensive database integrating genetic and environmental data, to explore these complex interactions and offer critical insights into the interplay between modifiable environmental factors and inherent genetic susceptibility. Specifically, this research aimed to investigate: (1) the association between changes in air pollutant exposure (PM_2.5_, NO_2_, sulfur dioxide [SO_2_], and ozone [O_3_]) and T2D risk; (2) the association of genetic predisposition, as measured by a PRS, on T2D risk; and (3) the additive interaction between air pollution and PRS in modulating T2D risk.

## Methods

### Data source and study population

The TWB is a longitudinal prospective cohort of 189,132 individuals aged 20 to 92 years recruited between March 2008 and December 2023, and each individual has been followed up since recruitment time [18]. Data on demographic and clinical characteristics, self-reported conditions, lifestyle conditions, and aging-related metrics were recorded using self-reported questionnaires at enrollment [19, 20]. All participants provided informed consent after receiving a thorough explanation of the study to ensure ethical compliance. This study was approved by the Institutional Review Board of the China Medical University (approval number: CRREC-108-006 (CR5)) and was conducted in accordance with the principals outlined in the Helsinki Declaration.

**Fig. 1:**
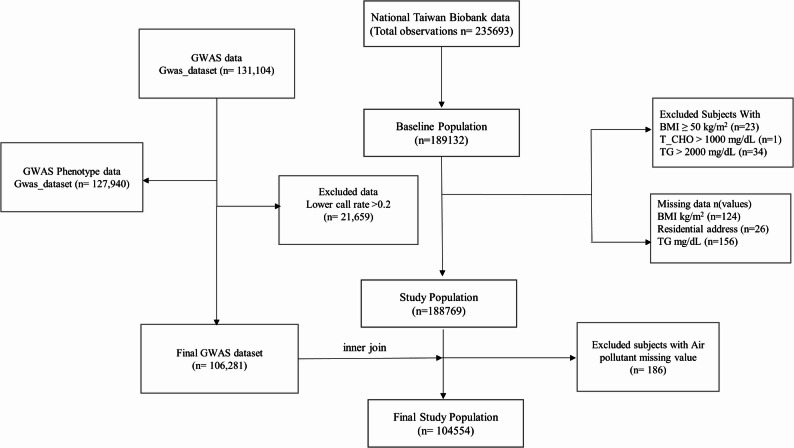
Flow chart of exclusion and inclusion criteria

This study included individuals aged 20–92 years from the TWB with available residential address data for geocoding air pollution exposure and genetic data for calculating PRS for T2D. Participants with missing in body mass index (BMI; *n* = 124) or residential address data (*n* = 26), outliers with extreme BMI (≥ 50 kg/m², *n* = 23), total cholesterol (> 1000 mg/dL, *n* = 1), or triglycerides (> 2000 mg/dL, *n* = 34), and without genotyping data (*n* = 84,029) were excluded. The flow chart was presented in the Fig. 1.

### Definition of outcome

The outcome, T2D, was defined based on self-reported medical conditions as recorded in the TWB health questionnaire. A previous published study demonstrated good-to-excellent concordance between self-reported T2D in the TWB and claims records from the National Health Insurance Research Database, with a tetrachoric correlation of 0.971 [19].

### Air pollution exposure assessment

Air pollution exposure data were derived from hybrid models integrated ground-based monitoring data, satellite observations, meteorological variables, and land-use data [21–24]. Air pollution data were sourced from 74 air quality monitoring stations across Taiwan, with hourly data from 2004 to 2022. Satellite-derived data included measurements from the Ozone Monitoring Instrument (OMI) aboard the Aura satellite and aerosol optical depth (AOD) from the Moderate Resolution Imaging Spectroradiometer (MODIS) sensors on Terra and Aqua satellites. Meteorological data, including temperature, wind components, precipitation, boundary layer height, and surface pressure, were obtained from ERA-5 reanalysis datasets. Land-use data were collected from the National Land Surveying and Mapping Center and the ESA Climate Change Initiative. Daily air pollution concentrations were estimated using eXtreme Gradient Boosting (XGBoost), achieving ten-fold cross-validation determination of coefficient (*R*^*2*^) of 0.90 for PM_2.5_, 0.79 for SO_2_, 0.82 for O_3_, and 0.92 for NO_2_, respectively. Daily concentrations were then averaged to derive annual concentrations.

To capture recent long-term exposure trends, we estimated five-year slopes of annual average concentrations of PM_2.5_, NO₂, SO₂, and O₃ prior to recruitment. For each participant, a linear regression model was fitted with yearly pollutant concentration regressed against time (Air Pollution_i_ = β₀ + β₁ × Time_i_ + ε_i_), where the coefficient β₁ (slope) represented the annual rate of change in pollutant exposure (µg/m³ per year). A positive slope indicated worsening air quality (i.e., increasing pollutant levels), where a negative slope indicated improvement [25]. We used pollutant slopes rather than traditional long-term average concentrations since air quality has generally improved across most regions of Taiwan in recent years [25]. While long-term average concentrations effectively capture spatial variation in exposure, they do not reflect temporal trends, an important limitation in a cross-sectional study. Therefore, pollutant slopes were used as the main exposure metric in this study to better characterize changes in air pollution.

### Genotyping and polygenic risk score (PRS)

A total of 131,104 participants were genotyped using the TWB v1 and TWB v2 SNP arrays (Thermo Fisher Scientific, Inc. Santa Clara, CA, USA). The two arrays share 104,463 markers. Imputation was performed using a population-specific reference haplotype panel from 1,445 genome sequences of the TWB [17]. Prior to conducting the GWAS analysis, stringent quality control (QC) procedures were applied. SNPs were excluded based on the following criteria: (1) individual or SNP missingness > 0.02, (2) minor allele frequency (MAF) < 0.05, (4) deviations from Hardy–Weinberg equilibrium (*p*-value < 1 × 10^− 10^ in cases and < 1 × 10^− 6^ in controls), (5) heterozygosity rates exceeding ± 3 standard deviations from the mean, (6) relatedness (pi-hat > 0.2), and (7) ethnic outliers, as defined by the principal component analysis [26].

The PRS was derived using summary statistics from the Asian Genetic Epidemiology Network (AGEN) T2D GWAS, which included East Asian cohorts [27]. This ancestry-specific dataset was chosen to ensure the transferability of the genetic risk model to the Taiwanese population. SNPs significantly associated with T2D in the AGEN GWAS were used as weights to calculate PRS values, which were then categorized into quartiles representing increasing genetic risk. The PRS was calculated using the formula:

$$PRS_i=\sum\limits_{j=1}^{n}\beta_j G_{ij}$$

where *n* is total number of SNPS; $\beta_j$is effect size of SNP j derived from GWAS summary statistics, which reflects the association between SNP*\:j* and T2D (expressed as a log odds ratio for binary traits such as T2D), and$\:{G}_{ij}$ is the genotype of individuals *j* at SNP *\:j*, reflecting the number of risk alleles (0, 1, or 2). To ensure independence among SNPs, clumping and thresholding (C + T) method was performed using PLINK 1.9 [28], with linkage disequilibrium (LD) pruning set at *r²* < 0.1 within a 250-kb window and *p*-value thresholds ranging from 5 × 10⁻⁸ to 0.5. The predictive performance of the PRS was evaluated using the ΔAUC (as compared with null model). The selection of the *p*-value threshold for PRS construction was based on the model that achieved the highest predictive performance (yielding the largest ΔAUC and pseudo-R²) across all tested thresholds (5 × 10⁻⁸ to 0.5). This data-driven approach follows standard PRS optimization practices used in previous studies employing the clumping and thresholding method [28, 29]. The final PRS score was computed based on 2,598,943 SNPs at a *p*-value threshold of 0.5, yielding moderate predictive ability (ΔAUC = 0.012). The top 20 most significant SNPs used in the PRS calculation are listed in Table S3.

PRS was categorized into quartiles: low genetic risk (< 25th percentile), moderate genetic risk (25th − 50th percentile), elevated genetic risk (50th − 75th percentile), and high genetic risk (> 75th percentile).

### Covariates

**Fig. 2:**
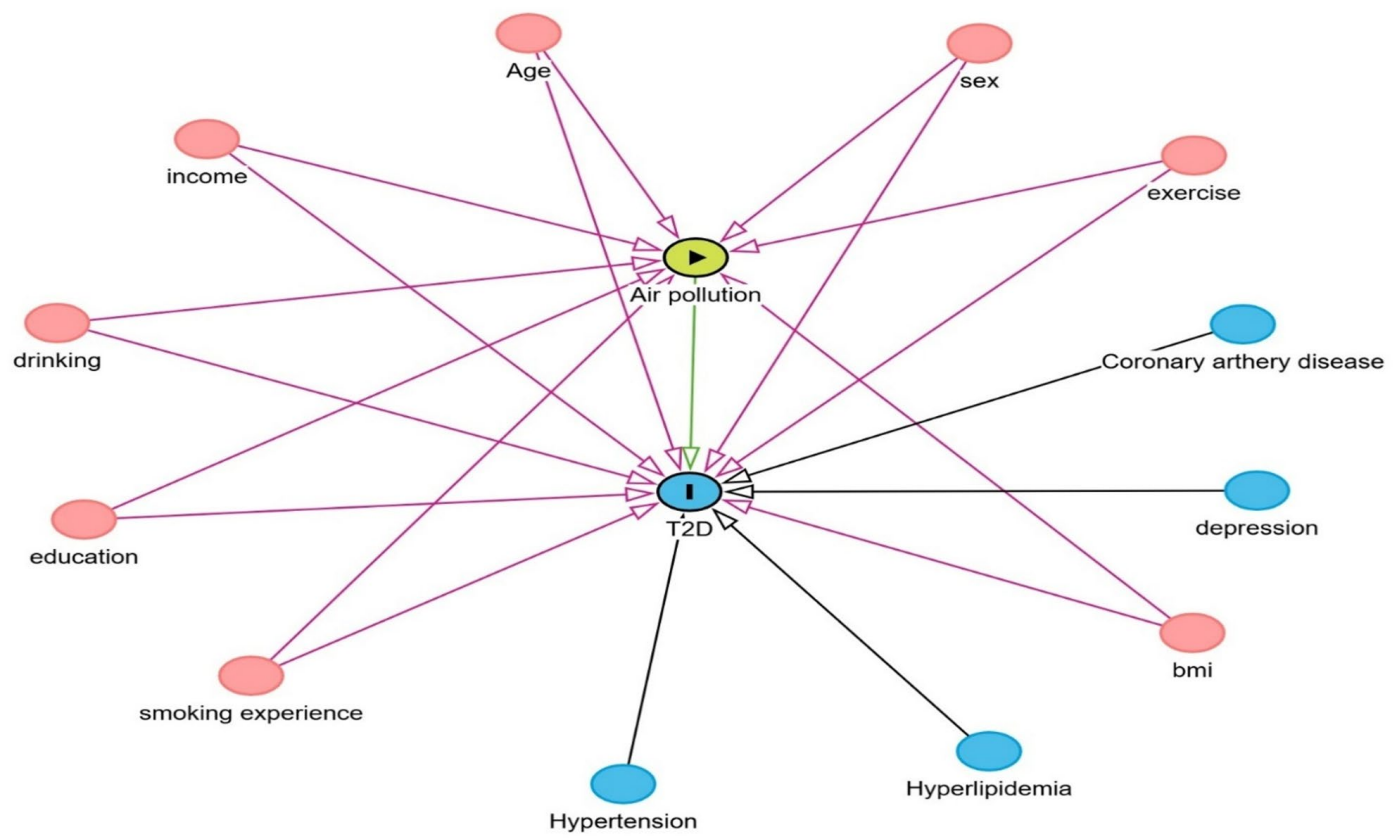
Directed acyclic diagram (DAG) diagram of the relationship between air pollution and type 2 diabetes mellitus (T2D) with confounders. The green node with arrow indicates the exposure (air pollutants), and the blue node with the Indicates the outcome of interest (T2D). Pink nodes represent adjusted confounders. Blue nodes are covariates that are associated with outcome but not with exposure

Potential cofounder variables were adjusted for in the analysis, including age (categorized into 20–39, 40–59, and 60–79 years) [30], sex (males and females) [31], and socioeconomic status was defined as individual monthly income (stratified into < NT$20,000, NT$20,000–NT$30,000, NT$30,000–NT$40,000, NT$40,000–NT$50,000, and > NT$50,000) [32]. BMI was categorized as underweight (< 18.5 kg/m^2^), normal (18.5–24.9 kg/m^2^), overweight (25–29.9 kg/m^2^), obesity I (30–34.9 kg/m^2^), obesity II (35–39.9 kg/m^2^), and obesity III (40–50 kg/m^2^) [33]. Education was classified into less than high school, high school, college/university, and postgraduate levels [34]. Key behavioral factors were also adjusted for: Exercise was categorized as a binary variable (“YES” or “NO”) based on self-reported data [35]; smoking status was classified as never, former, or current smokers [36].Finally, major comorbidities were included as binary variables: hypertension, hyperlipidemia, and depression. Given that these factors are established risk factors for T2D, all were systematically adjusted for in the model to mitigate potential confounding bias (Fig. 2).

### Statistical analysis

Chi-square tests were conducted to assess the associations between categorical variables and T2D. Univariate logistic regressions were performed to evaluate the relationships between individual covariates and T2D. Multivariable logistic regression models were then employed to assess the association of T2D with air pollution exposure, including NO_2_, O_3_, PM_2.5_, and SO_2_, as well as with PRS. Exposure was represented by long-term air pollutant trends, quantified as slopes. Model 1 was adjusted for age, sex, family income, BMI, education, exercise, smoking status, and drinking status (Fig. 2). As a sensitivity analysis, Model 2 included all covariates in in Model 1, with additional adjustment for comorbidities such as hypertension, depression, and hyperlipidemia. Results were presented as odds ratios (ORs) with corresponding 95% confidence intervals (CIs).

Additive interaction between PRS categories and air pollution exposure was assessed using the RERI, calculated as:

$$\mathrm{RERI} = exp (\beta1+\beta2+\beta3) -exp(\beta1) -exp(\beta2)+1$$

where $\beta1,\beta2,\beta3$ represent coefficients for the pollutant slope, a given PRS category, and their interaction term, respectively. Bootstrapping with 1,000 iterations was performed to derive 95% confidence intervals for RERI [37]. Additionally, multiplicative interactions were also tested by including cross-product terms between air pollutant slopes and PRS categories in multivariable logistic regression models. The statistical significance of the interaction terms was evaluated using the Wald test. All analyses were conducted using R (version 4.4.2, R Core Team), employing the following packages: magrittr, dplyr, tidyverse, ggplot2, and boot.

## Results

### Demographic characteristics and comorbidities of study population

Of the 104,554 individuals included in the study, 5,315 (5.08%) were identified as having T2D, with prevalence varying markedly across demographics and health-related factors. Order age, lower education attainment, lower socioeconomic status (as indicated by monthly income), higher BMI, former and current smoking, occasional alcohol consumption, regular exercise habit, and major comorbidities, including hypertension, hyperlipidemia, and depression, were all positively associated with an increased risk of T2D (Table 1).

**Table 1:** Demographic characteristics and comorbidities of study population (*n* = 104,554)

Variables	Diabetes cases *N* (%) 5,315 (5.08)	Non-diabetes *N* (%) 99,239 (94.92)	Odds ratio (95% CI)	*p*-value^a^
**Age (years)**				< 0.001
20–39	240 (4.51)	22,741 (22.91)	Reference	
40–59	2,606 (49.03)	55,908 (56.33)	4.41 (3.86, 5.04)	
60–79	2,469 (46.45)	20,590 (20.74)	11.36 (9.93, 12.99)	
**Gender**				< 0.001
Male	2,542 (47.82)	35,285 (35.55)	1.66 (1.57, 1.76)	
Female	2,773 (52.17)	63,954 (64.44)	Reference	
**Education**				< 0.001
Lower than high school	1,176 (22.12)	11,073 (11.15)	2.49 (2.31, 2.68)	
High school	1,728 (32.51)	28,478 (28.69)	1.42 (1.33, 1.52)	
College or university	2,053 (38.62)	48,150 (48.51)	Reference	
Postgraduate	358 (06.73)	11,538 (11.62)	0.72 (0.64, 0.81)	
**BMI**				< 0.001
Underweight (≤ 12–18.5)	66 (1.24)	3,279 (3.29)	0.54 (0.42, 0.70)	
Normal (18.50–24.9)	2,213 (41.63)	60,238 (60.69)	Reference	
Overweight (25–29.9)	2,159 (40.57)	28,976 (29.14)	2.03 (1.91, 2.15)	
Obesity I (30–34.9)	669 (12.58)	5,751 (5.79)	3.16 (2.89, 3.46)	
Obesity II (35–39.9)	170 (3.19)	882 (0.88)	5.24 (4.41, 6.21)	
Obesity III (40–50)	40 (0.75)	178 (0.17)	2.03 (1.91,2.16)	
**Individual Monthly Income**				< 0.001
< NT$ 20,000	3,295 (61.99)	53,665 (54.07)	Reference	
NT$ 20,000–30,000	838 (15.76)	18,552 (18.69)	0.73 (0.68, 0.79)	
NT$ 30,000–40,000	1140 (21.44)	26,383 (26.58)	0.70 (0.65, 0.75)	
> NT$ 40,000	42 (0.79)	639 (0.64)	1.07 (0.78, 1.46)	
**Drinking status**				< 0.001
Quit	4,618 (86.88)	90,937 (91.63)	0.38 (0.34, 0.43)	
Occasional	323 (6.07)	2,445 (2.46)	Reference	
Regular	374 (7.03)	5,857 (5.90)	0.48 (0.41, 0.57)	
**Smoking status**				< 0.001
Never	3,785 (71.21)	80,163 (80.77)	Reference	
Former	897 (16.87)	9,880 (9.95)	1.92 (1.78, 2.07)	
Current	633 (11.90)	9,196 (9.26)	1.45 (1.33, 1.59)	
**Exercise habit**				< 0.001
Yes	2,578 (48.50)	39,207 (39.50)	1.44 (1.36, 1.52)	
No	2,737 (51.49)	60,032 (60.49)	Reference	
**Hypertension**				< 0.001
Yes	2,186 (41.12)	10,375 (10.45)	5.98 (5.64, 6.34)	
No	3,129 (58.87)	88,864 (89.54)	Reference	
**Hyperlipidemia**				< 0.001
Yes	1,744 (32.81)	6,104 (6.15)	7.45 (6.99, 7.93)	
No	3,571 (67.18)	93,135 (93.84)	Reference	
**Depression**				< 0.001
Yes	295 (5.55)	3,516 (3.54)	1.60 (1.41, 1.80)	
No	5,020 (94.44)	95,723 (96.45)	Reference	

^a^Chi-square test was used

^b^*p* < 0.005

### Descriptive statistics of air pollutants of slopes

Table 2 presents the descriptive statistics of air pollutant slopes, representing annual changes in concentrations. The mean slopes (± SD) were as follows: PM_2.5_ (− 1.56 ± 0.88), NO_2_ (− 0.44 ± 0.59), SO_2_ (− 0.13 ± 0.13), and O_3_ (− 0.01 ± 0.61). Median values for the slopes were − 1.37 for PM_2.5_, − 0.337 for NO_2_, − 0.11 for SO_2_, and 0.00 for O_3_. All air pollutants in Taiwan showed a deceasing trend, except for O_3_.

**Table 2:** Descriptive statistics of baseline concentrations and five-year slope characteristics of ambient air pollutants, including particulate matter with an aerodynamic diameter < 2.5 μm (PM_2.5_), nitrogen dioxide (NO_2_), sulfur dioxide (SO_2_), and Ozone (O_3_)

Year	Mean	SD	Median	Min	Max	Q1	Q3	IQR
Baseline PM_2.5_	26.7	7.64	25.3	6.40	62.6	21.0	32.7	11.7
PM_2.5_ Slope	-1.56	0.88	-1.37	-5.01	0.42	-2.05	-0.90	1.15
Baseline NO_2_	14.3	6.39	13.6	2.13	36.4	9.47	17.5	8.06
NO_2_ Slope	-0.44	0.59	-0.337	-4.71	2.03	-0.60	-0.16	0.44
Baseline SO_2_	3.34	0.95	3.13	1.19	9.82	2.69	3.74	1.05
SO_2_ Slope	-0.13	0.13	-0.11	-0.80	0.39	-0.20	-0.03	0.17
Baseline O_3_	45.4	4.33	45.8	30.8	60.9	42.7	48.2	5.53
O_3_ Slope	-0.01	0.61	-0.00	-2.62	2.74	-0.37	0.34	0.71

IQR, interquartile range; Max, maximum; Min, minimum; Q1, 25 percentiles; Q3, 75 percentile; SD, standard deviation

### Association between air pollution and T2D

The results of logistic regressions are shown in Table 3. After controlling for potential confounding factors, the PM_2.5_ slope was significantly positively associated with T2D. The adjusted OR for every 1 µg/m^3^ per year increase in PM_2.5_ level over the five years preceding recruitment was 1.036 (95% CI: 1.003–1.071), indicating that worsening PM_2.5_ exposure was associated with higher odds of T2D. In contrast, no significant associations were observed for changes in NO_2_, SO_2_, and O_3_ levels.

**Table 3:** Associations of slopes in air pollutants with type 2 diabetes mellitus (T2D), presented as odds ratios (ORs) and 95% confidence interval (CI)

Air pollutants	Crude Model OR (95% CI)	Adjusted Model 1 OR (95% CI)	Adjusted Model 2 OR (95% CI)
PM_2.5_ slope	1.032 (1.000, 1.065)*	1.036 (1.003, 1.071)*	1.048 (1.012, 1.084)*
NO_2_ slope	0.979 (0.935, 1.025)	0.970 (0.925, 1.018)	0.972 (0.925, 1.022)
SO_2_ slope	0.996 (0.810, 1.223)	0.854 (0.691, 1.056)	0.875 (0.704, 1.088)
O_3_ slope	1.035 (0.990, 1.083)	1.005 (0.959, 1.053)	0.991 (0.944, 1.040)

CI, confidence interval; NO_2_, nitrogen dioxide; O_3_, ozone; OR, odds ratio; PM_2.5_, particulate matter with an aerodynamic diameter < 2.5 μm

Model 1 adjusted for age, sex, individual monthly income, BMI, education, exercise, smoking status, and drinking status

Model 2 adjusted for age, sex, individual monthly income, BMI, education, exercise, smoking status, drinking status, hypertension, depression and hyperlipidemia

*p < 0.05

Multiple pollutant models were employed to account for interactions and combined association of pollutants. The results confirmed the robust association of every 1 g/m^3^ per year increase in PM_2.5_ with T2D when considering the other air pollutants (Table S1).

### Associations between PRS and T2D

The associations between PRS and T2D are presented in Table 4. A clear dose–response relationship was observed, with higher PRS quartiles associated with progressively increased odds of T2D compared with the lowest quartile. In the fully adjusted model (Model 1), ORs were 1.121 (95% CI: 1.033–1.217) for Q2, 1.136 (95% CI: 1.046–1.233) for Q3, and 1.385 (95% CI: 1.279–1.499) for Q4. Additional adjustment for prevalent comorbidities, including hypertension, hyperlipidemia, and depression (Model 2), yielded similar estimates (Q4 OR: 1.382, 95% CI: 1.273–1.500). These consistent results indicate that genetic susceptibility, as quantified by the PRS, was robustly associated with T2D risk independent of lifestyle and clinical factors.

**Table 4:** Associations between polygenic risk scores (PRS) and type 2 diabetes mellitus (T2D), presented as odds ratio (OR) and 95% confidence interval (CI)

PRS Category (Quartile Range)	Crude Model OR (95% CI)	Adjusted Model 1 OR (95% CI)	Adjusted Model 2 OR (95% CI)
Q1 (0.5092–0.5143)	Reference	Reference	Reference
Q2 (0.5143–0.5153)	1.109 (1.023,1.202)*	1.121 (1.033,1.217)*	1.113 (1.023,1.211)*
Q3 (0.5153–0.5163)	1.131 (1.044,1.226)*	1.136 (1.046,1.233)*	1.119 (1.028,1.217)*
Q4 (0.5163–0.5213)	1.369 (1.266,1.479)*	1.385 (1.279,1.499)*	1.382 (1.273,1.500)*

Model 1 adjusted for age, sex, individual monthly income, BMI, education, exercise, smoking status, and drinking status

Model 2 adjusted for age, sex, individual monthly income, BMI, education, exercise, smoking status, drinking status, hypertension, depression and hyperlipidemia

*p < 0.05

### Interactions of air pollution and PRS with T2D

Table 5 summarizes the associations between air pollutant slopes and T2D risk across PRS quartiles, with PRS category 4 representing the highest genetic predisposition. The ORs for T2D per 1 µg/m^3^ increase in PM_2.5_ level per year for the lowest (quartile 1) to the highest genetic risk group (quartile 4) were 0.993 (95% CI: 0.936,1.054 ), 1.027 (95% CI: 0.960,1.098 ), 1.045 (95% CI: 0.976,1.12 ), and 1.097 (95% CI: 1.022,1.178), respectively (Table 5). A weak and marginal additive interaction was identified in the highest genetic risk group (RERI: 0.144, 95% CI: 0.008, 0.319). For NO_2_, SO_2_, and O_3_ slopes, no significant association with T2D was identified across any PRS category. The ORs of T2D ranged from 0.952 to 0.976 for NO_2_, from 0.663 to 0.994 for SO_2_, and from 0.995 to 0.986 for O_3_. All RERI values were statistically insignificant, suggesting minimal evidence of additive interaction between these gaseous pollutants and genetic susceptibility on T2D. Furthermore, none of the multiplicative interaction terms achieved statistical significance (Table S2). These results demonstrate that worsening PM_2.5_ exposure was significantly associated with T2D among individuals with high genetic susceptibility on the additive scale, while other pollutants, including NO_2_, SO_2_, and O_3_, do not exhibit significant associations or interactions with T2D.

**Table 5:** Associations of slopes in air pollutants with type 2 diabetes mellitus categorized by polygenic risk score (PRS), presented as odds ratios (ORs) and 95% confidence interval (CI)

Air pollutants	PRS < Q1	Q1–Q2	Q2–Q3	> Q3
	OR (95% CI)^a^	OR (95% CI)	OR (95% CI)	OR (95% CI)
PM_2.5_ slope (− 5.01–0.42)	0.993 (0.936,1.054)	1.027 (0.960,1.098)	1.045 (0.976,1.12)	**1.097** **(1.022**,1.178)*
RERI (95% CI)	-	0.024 (-0.067, 0.121)	0.051 (-0.043, 0.175)	**0.144** **(0.008**,** 0.319)**
NO_2_ slope (− 4.71– 2.03)	0.952 (0.873,1.040)	0.920 (0.838,1.011)	1.043 (0.938,1.16)	0.976 (0.882,1.080)
RERI (95% CI)	-	-0.030 (-0.155, 0.103)	0.092 (-0.057, 0.269)	0.009 (-0.143, 0.206)
SO_2_ slope (− 0.80–0.39)	0.663 (0.455,0.967)	0.799 (0.521,1.226)	0.980 (0.626,1.535)	0.994 (0.634,1.557)
RERI (95% CI)	-	0.158 (-0.253, 0.709)	0.306 (-0.223, 1.052)	0.348 (-0.239, 1.340)
O_3_ slope (− 2.62–2.74)	0.995 (0.914,1.084)	1.053 (0.956,1.160)	0.985 (0.893,1.088)	0.986 (0.893,1.089)
RERI (95% CI)	-	0.060 (-0.083, 0.209)	-0.008 (-0.151, 0.134)	-0.012 (-0.166, 0.166)

CI, confidence interval; NO_2_, nitrogen dioxide; O_3_, ozone; OR, odds ratio; PM_2.5_, particulate matter with an aerodynamic diameter < 2.5 μm

Models adjusted for age, sex, individual monthly income, body mass index (BMI), education, exercise, smoking status, and drinking status

*p < 0.05

## Discussion

This study assessed the independent and joint association of air pollution exposure and genetic susceptibility, as measured by PRS, on T2D risk using TWB data. Consistent with previous literature, long-term exposure to worsening PM_2.5_ was associated with T2D (adjusted OR: 1.036; 95% CI: 1.003–1.071) [36, 38]. Our findings also revealed a clear exposure-response relationship between PRS and T2D, indicating that individuals in highest PRS category had 38.5% higher risk relative to those in lowest quartile. The association between PM_2.5_ and T2D was stronger in individuals with the high PRS score (OR: 1.097, 95% CI: 1.022–1.178), although the additive interaction was minimal (RERI: 0.144, 95% CI: 0.008–0.319). These results highlight the multifactorial nature of T2D and the need for integrated strategies addressing both genetic susceptibility and environmental exposures to mitigate T2D risk [10].

Increasing in PM_2.5_ slope exhibited a statistically significant association with T2D, aligned with existing literature reporting a 10–28% associated with higher odds per 10 µg/m³ increment in long-term PM_2.5_ exposure [7, 39]. Our findings are parallel to previous finding, as every 1 µg/m^3^ increase in PM_2.5_ per year was significantly associated with T2D (OR: 1.036, 95% CI: 1.003–1.071). Mechanistically, PM_2.5_ has been shown to trigger systemic inflammation, oxidative stress, and insulin resistance, which are key contributors to diabetes pathogenesis [9, 40]. This study’s findings align with these mechanisms, reinforcing the role of PM_2.5_ as a critical environmental risk factor for T2D.

The stratified analysis identified a signal for a potential gene-environment interaction, as the association between PM_2.5_ and T2D was maximum in participants within the highest PRS quartile (OR: 1.097, 95% CI: 1.022–1.178). However, the evidence for additive interaction was weak and marginal (RERI: 0.144, 95% CI: 0.008–0.319). Nevertheless, this relationship is supported by existing literature, including a Korean study that demonstrated the modification of T2D risk by PRS in conjunction with environmental factors such as waist circumference [41]. Similarly, a China study showed that healthy lifestyles attenuated genetic risk for T2D in Chinese adults, emphasizing the modifiable nature of environmental exposures even among those with high PRS [42]. Large Western cohorts, such as the UK Biobank, where long-term PM_2.5_ exposure was linked to elevated T2D incidence and stronger pattern among those with high genetic risk [10]. Similarly, a Swiss cohort study demonstrated that PM_10_ exposure interacted with a T2D genetic risk score to elevate diabetes risk [43].

In contrast, NO_2_, SO_2_, and O_3_ were not significantly associated with T2D in both crude or adjusted models, prior studies have reported mixed and often inconsistent findings. For instance, one cohort study found no significant association between NO₂ exposure and incident T2D after adjusting for covariates, while SO₂ and O₃ were only weakly associated with dysglycemia markers like impaired fasting glucose, but not full T2D [44]. Furthermore, a recent U.S. study analyzing three large cohorts demonstrated no obvious associations between PM_2.5_, O₃, and the incident T2D after adjusting for covariates, emphasizing the absence of a consistent relationship between PM_2.5_, O₃, and T2D [45]. The lack of associations for NO_2_ and O_3_ may reflect their lower concentrations or indirect metabolic effects, while reductions in SO_2_ levels, likely due to regulatory interventions, may have minimized its health impact [46]. Additionally, PM_2.5_ differs from gaseous pollutants in its ability to penetrate deep into the alveoli and enter systemic circulation, where it promotes oxidative stress, inflammation, and insulin resistance, whereas NO_2_, SO_2_, and O_3_ mainly act on the respiratory epithelium with limited systemic effects [9, 47–49]. These mechanistic differences may partly explain why PM_2.5_ showed a stronger and more consistent association with T2D compared to gaseous pollutants.

We found a significant association between PRS and T2D susceptibility. Individuals in the highest PRS quartile exhibited a 38.5% elevation in risk relative to the lowest quartile (Q1) (Model 1: OR:1.385, 95% CI: 1.279–1.499). This dose-response relationship is consistent with results from major multi-ethnic cohorts, which have uniformly demonstrated a significant positive correlation between placement in the highest PRS categories and T2D incidence [50]. Our PRS was tailored to maximize relevance by leveraging GWAS summary statistics from the Asian Genetic Epidemiology Network (AGEN) [27]. The robustness of the observed associations across multiple PRS constructs validates the methodology and points toward the potential clinical utility of genetic risk stratification for targeted environmental and lifestyle interventions. Ultimately, these results reiterate the scientific mandate for developing population-specific PRS models to achieve enhanced predictive accuracy and better inform personalized medicine strategies within Asian populations [42].

A stronger significant association between PM_2.5_ slope and T2D was found in the highest genetic risk group (OR: 1.097, 95% CI: 1.022 to 1.022,1.178); however, the additive interaction between PM_2.5_ and PRS was borderline significant, with RERI = 0.144 and 95% CI: 0.008 to 0.319, suggesting a possible but inconclusive combined association. Additionally, multiplicative interaction analyses were not statistically significant (Table S2). Compared to the multiplicative scale, the additive interaction was prioritized because it provides a more biologically and public health–relevant measure of the combined association of both genetic and environmental exposures on the absolute risk scale [51]. The findings emphasize the cumulative risk of T2D among individuals exposed to both high genetic and environmental factors [10]. Mechanistically, prolonged exposure of PM_2.5_ has been associated to epigenetic modifications and altered gene expression in pathways critical to insulin signaling, potentially heightening diabetes risk in genetically susceptible individuals [52]. The pronounced association of PM_2.5_ may be explained by its capacity to induce oxidative stress and systemic inflammation, exacerbating β-cell dysfunction and insulin resistance through interactions with genetic pathways [53]. These findings reinforce the need for integrated assessments that account for both genetic predispositions and environmental exposures.

This study possesses significant strengths. The large sample size of 104,554 participants and the availability of detailed demographic, genetic, and environmental data provide robust statistical power and enhance generalizability. Additionally, this research is among the first in Eastern Asia to explore the interplay between PRS and air pollution trends, offering critical insights into the joint relationship of genetic predisposition and environmental factors on T2D risk. The use of slopes for air pollutant trends adds a dynamic temporal perspective, distinguishing this study from others that rely on static exposure data.

However, this study has several limitations. First, T2D status was based on self- reported physician diagnosis, and the potential misclassification cannot be completely excluded. Nevertheless, a validation study comparing TWB data with the National Health Insurance Research Database demonstrated excellent concordance between self-reported and registry-based diagnoses (tetrachoric correlation = 0.971) [19]. While a small degree of misclassification may persist, it is likely to be non-differential with respect to T2D case and non-case groups. Therefore, any resulting bias would likely be attenuate the observed associations toward the null and is unlikely to have substantially influenced our findings. Second, although we incorporated five-year air pollution exposure trends preceding recruitment, thereby enhancing temporal alignment compared with simple cross-sectional designs, the study remains observational, and causal inference is limited. Future longitudinal or quasi-experimental studies, including natural experiments, are warranted to strengthen the causal inference [51, 54, 55]. Third, as a cross-sectional study, our analysis remains susceptible to reverse causality. For example, we observed that regular exercise was associated with a higher risk of T2D, which may appear paradoxical (Table 1). This likely suggests reverse causation, wherein individuals previously diagnosed with or at higher risk of diabetes deliberately increase their physical activity in accordance with medical advice. Similar paradoxical trends have been observed in other large biobank and cohort studies, where lifestyle modification after diagnosis leads to cross-sectional associations that imply a higher disease prevalence among physically active individuals [56, 57]. In addition, the association may also be influenced by residual confounding from unmeasured variables such as diet quality, exercise intensity, and duration. Prospective longitudinal studies are needed to clarify the temporal relationship between lifestyle behaviors and T2D risk.

## Conclusion

This study addressed a potential additive interaction between air pollution exposure and genetic predisposition in T2D within a large Taiwanese population. PRS emerged as a significant predictor of T2D, emphasizing the need for ethnicity-specific models to improve predictive accuracy. PM_2.5_ slope was significantly associated with T2D, while other pollutants showed no significant associations. The observed interaction between PM_2.5_ slope and PRS was weak and borderline significant, suggesting a possible synergistic effect that should be interpreted cautiously and validated in longitudinal or mechanistic studies. These findings advocate for integrated genetic-environmental approaches to better understand T2D risk. Reducing PM_2.5_ exposure and incorporating genetic screening into clinical care could enable targeted prevention, particularly for high-risk groups. Future research should focus on developing ancestry-specific PRS models and clarifying the biological pathways linking pollution to T2D, supporting precision medicine and informed public health policies.

## Supplementary Information

Below is the link to the electronic supplementary material.

Supplementary Material 1

## Data Availability

The data that support the findings of this study are available from the Ministry of Health and Welfare, Taiwan but restrictions apply to the availability of these data, which were used under license for the current study, and so are not publicly available. Data are however available from the authors upon reasonable request and with permission of the Ministry of Health and Welfare, Taiwan.

## Abbreviations

AOD: Aerosol optical depth
AUC: Area under the curve
C+T: Clumping and thresholding method
CI: Confidence interval
CRS: Clinical risk score
GWAS: Genome wide association studies
HR: Hazard ratio
IDF: International Diabetes Federation
MAF: Minor allele frequency
NO$_2$: Nitrogen dioxide
O$$_3$$: Ozone
OMI: Ozone monitoring instrument
OR: Odds ratio
PM$_{2.5}$: Fine particulate matter
R$^2$: Coefficient of determination
RERI: Relative excess risk due to interaction
PRS: Polygenic risk scores
QC: Quality control
SES: Socioeconomic status
SNP: Single nucleotide polymorphism
SO$_2$: Sulfur dioxide
T2D: Type 2 diabetes mellitus
TWB: Taiwan Biobank
WHO: World Health Organization
XGBoost: eXtreme Gradient Boosting
