# Correction: Polygenic risk and air pollution trends in relation to type 2 diabetes: evidence from the Taiwan Biobank

**DOI:** 10.1186/s13098-026-02263-4

**Published:** 2026-08-04

**Authors:** Osama Aziz, Bing-Fang Hwang, Ai-Ru Hsieh, Chau-Ren Jung

**Affiliations:** 1 Department of Public Health, College of Public Health, China Medical University, No. 100, Sec. 1, Jingmao Rd. Beitun Dist, Taichung, 406040 Taiwan; 2 Department of Occupational Safety and Health, College of Public Health, China Medical University, Taichung, Taiwan; 3 Department of Statistics, National Taipei University, No.151, University Rd., San Shia District, New Taipei, 23741 Taiwan; 4 Japan Environment and Children’s Study Programme Office, National Institute for Environmental Studies,, Tsukuba, Japan

**Correction : Diabetol Metab Syndr (2026) 18:64**

10.1186/s13098-026-02088-1

In article [1], the authors identified a error in the formula given in the paragraph “Statistical analysis” which comes under “Methods” section in the article.

Incorrect Formula:

RERI = exp(β1 + β2 + β3) − exp(β2) + 1.

Correct Formula:

RERI = exp(β1 + β2 + β3) − exp(β1) − exp(β2) + 1.

The original article has been corrected.
