# Supplementary material for: Polygenic risk and air pollution trends in relation to type 2 diabetes: evidence from the Taiwan Biobank
Source: Diabetol Metab Syndr. 2026 Jan 20;18:64. doi: 10.1186/s13098-026-02088-1 (PMC12905863; doi:10.1186/s13098-026-02088-1)
Supplement: Supplementary file 1 — Supplementary Material 1 [file 13098_2026_2088_MOESM1_ESM.docx]

Supplementary Materials

**Polygenic Risk and Air Pollution Trends in Relation to Type 2 Diabetes: Evidence from the Taiwan Biobank**

Osama Aziz^a^, Bing-Fang Hwang^b,c^, Ai-Ru Hsieh^d,*^, Chau-Ren Jung^a,e,*^

**Affiliations:**

^a^Department of Public Health, College of Public Health, China Medical University, Taichung, Taiwan

^b^Department of Occupational Safety and Health, College of Public Health, China

Medical University, Taichung, Taiwan

^c^Department of Occupational Therapy, College of Medical and Health Science, Asia University, Taichung, Taiwan

^d^Department of Statistics, National Taipei University, New Taipei City, Taiwan

^e^Japan Environment and Children’s Study Programme Office, National Institute for Environmental Studies, Tsukuba, Japan

**Table of Contents**

**Table S1**. Multiple pollutant models for slopes of air pollutants and type 2 diabetes mellitus, presented as odds ratio (OR) and 95% confidence interval (CI).

**Table S2.** Multiplicative interaction between air pollutant slope and polygenic risk score (PRS) categories on type 2 diabetes mellitus (T2D) risk, expressed as odds ratios (ORs), 95% confidence intervals (CI) and p for interaction.

**Table S3.** The list of top 20 significant single nucleotide polymorphisms (SNPs) associated with type 2 diabetes mellitus (Based on P-value).

**Table S1**. Multiple pollutant models for slopes of air pollutants and type 2 diabetes mellitus (T2D), presented as odds ratio (OR) and 95% confidence interval (CI).

|  |  | Crude |  | Adjusted |  |
| --- | --- | --- | --- | --- | --- |
| Model | Pollutant | Odds ratio | 95% CI | Odds ratio | 95% CI |
| PM_2.5_ slope+NO_2_ slope | PM_2.5_ slope | 1.032 | (1.000,1.065) | 1.037 | (1.003,1.072) |
|  | NO_2_ slope | 0.979 | (0.935,1.025) | 0.969 | (0.924,1.016) |
| PM_2.5_ slope+SO_2_ slope | PM_2.5_ slope | 1.032 | (1.000,1.065) | 1.044 | (1.010,1.081) |
|  | SO_2_ slope | 0.996 | (0.810,1.223) | 0.803 | (0.647,0.998) |
| PM_2.5_ slope+ O_3_ slope | PM_2.5_ slope | 1.032 | (1.000,1.065) | 1.037 | (1.003,1.072) |
|  | O_3_ slope | 1.035 | (0.990,1.065) | 0.996 | (0.950,1.042) |
| NO_2_ slope+ SO_2_ slope | NO_2_ slope | 0.979 | (0.935,1.025) | 0.975 | (0.928,1.023) |
|  | SO_2_ slope | 0.996 | (0.810,1.223) | 0.869 | (0.701,1.077) |
| NO_2_ slope+ O_3_ slope | NO_2_ slope | 0.979 | (0.935,1.025) | 0.969 | (0.921,1.019) |
|  | O_3_ slope | 1.035 | (0.990,1.083) | 0.995 | (0.947,1.045) |
| SO_2_ slope+O_3_ slope | SO_2_ slope | 0.996 | (0.810,1.223) | 0.855 | (0.692,1.056) |
|  | O_3_ slope | 1.035 | (0.990,1.083) | 1.004 | (0.958,1.053) |

Abbreviations: CI, confidence interval; NO_2_, nitrogen dioxide; O_3_, ozone; OR, odds ratio; PM_2.5_, particulate matter with an aerodynamic diameter < 2.5μm.

Notes: Models adjusted for age, sex, family income, BMI, education, exercise, smoking status, and drinking status

*p < 0.05

**Table S2**. Multiplicative interaction between air pollutant slope and polygenic risk score (PRS) categories on type 2 diabetes mellitus (T2D) risk, expressed as odds ratios (ORs), 95% confidence intervals (CI) and p for interaction.

| Air pollutants | Adjusted |  | *p* for interaction |
| --- | --- | --- | --- |
|  | Odds ratio | 95% CI |  |
| PM_2.5_ slope |  |  |  |
| PRS < Q1 | 0.993 | (0.936, 1.054) | - |
| Q1–Q2 | 1.027 | (0.960, 1.098) | 0.857 |
| Q2–Q3 | 1.045 | (0.976, 1.120) | 0.909 |
| **> Q3** | **1.097** | **(1.022, 1.178)** | 0.206 |
| NO_2_ slope |  |  |  |
| PRS < Q1 | 0.952 | (0.873,1.040) | - |
| Q1–Q2 | 0.920 | (0.838,1.011) | 0.463 |
| Q2–Q3 | 1.043 | (0.938,1.160) | 0.965 |
| > Q3 | 0.976 | (0.882,1.080) | 0.311 |
| SO_2_ slope |  |  |  |
| PRS < Q1 | 0.663 | (0.455,0.967) | - |
| Q1–Q2 | 0.799 | (0.521,1.226) | 0.434 |
| Q2–Q3 | 0.980 | (0.626,1.535) | 0.517 |
| > Q3 | 0.994 | (0.634,1.557) | 0.183 |
| O_3_ slope |  |  |  |
| PRS < Q1 | 0.995 | (0.914,1.084) | - |
| Q1–Q2 | 1.053 | (0.956,1.160) | 0.911 |
| Q2–Q3 | 0.985 | (0.893,1.088) | 0.914 |
| > Q3 | 0.986 | (0.893,1.089) | 0.510 |

Abbreviations: CI, confidence interval; NO_2_, nitrogen dioxide; O_3_, ozone; OR, odds ratio; PM_2.5_, particulate matter with an aerodynamic diameter < 2.5μm; SO_2_, sulfur dioxide.

Notes: Models adjusted for age, sex, socioeconomic status, body mass index (BMI), education, exercise, smoking status, and drinking status.

**Table S3.** The list of top 20 significant single nucleotide polymorphisms (SNPs) associated with type 2 diabetes mellitus (Based on P-value).

| **SNP** | **CHR** | **POS** | **EFF_ALLELE** | **NONEFF_ALLELE** | **EFF_ALLELE_FREQ** | **BETA** | **SEBETA** | **P-value** |
| --- | --- | --- | --- | --- | --- | --- | --- | --- |
| rs11049939 | 12 | 29,100,629 | A | G | 0.0000 | -1.5708 | 0.1817 | 5.27 × 10⁻¹⁸ |
| rs2237896 | 11 | 2858440 | A | G | 0.3740 | -0.2452 | 0.0288 | 1.59 × 10⁻¹⁷ |
| rs7754840 | 6 | 20661250 | C | G | 0.4008 | 0.2044 | 0.0240 | 1.86 × 10⁻¹⁷ |
| rs7772603 | 6 | 20665946 | T | C | 0.5992 | -0.2172 | 0.0256 | 2.19 × 10⁻¹⁷ |
| rs4710940 | 6 | 20658012 | A | C | 0.5992 | -0.2114 | 0.0249 | 2.20 × 10⁻¹⁷ |
| rs9460546 | 6 | 20663632 | T | G | 0.6002 | -0.2116 | 0.0250 | 2.37 × 10⁻¹⁷ |
| rs4712522 | 6 | 20656800 | C | G | 0.5992 | -0.2109 | 0.0249 | 2.66 × 10⁻¹⁷ |
| rs6456367 | 6 | 20659587 | A | T | 0.4008 | 0.2103 | 0.0249 | 2.81 × 10⁻¹⁷ |
| rs7774594 | 6 | 20661143 | A | T | 0.4008 | 0.2103 | 0.0249 | 3.33 × 10⁻¹⁷ |
| rs9460544 | 6 | 20661529 | T | G | 0.4008 | 0.2102 | 0.0249 | 3.40 × 10⁻¹⁷ |
| rs9460545 | 6 | 20661550 | T | C | 0.5992 | -0.2101 | 0.0249 | 3.58 × 10⁻¹⁷ |
| rs10946398 | 6 | 20661034 | A | C | 0.5992 | -0.2096 | 0.0249 | 3.79 × 10⁻¹⁷ |
| rs9358356 | 6 | 20667382 | T | C | 0.5992 | -0.2098 | 0.0250 | 4.21 × 10⁻¹⁷ |
| rs7752780 | 6 | 20666022 | A | G | 0.4008 | 0.2093 | 0.0249 | 4.72 × 10⁻¹⁷ |
| rs6906327 | 6 | 20659459 | A | G | 0.3998 | 0.2010 | 0.0240 | 5.33 × 10⁻¹⁷ |
| rs4712525 | 6 | 20662966 | T | C | 0.4008 | 0.2080 | 0.0249 | 7.17 × 10⁻¹⁷ |
| rs4712526 | 6 | 20663035 | A | T | 0.4008 | 0.2079 | 0.0249 | 7.53 × 10⁻¹⁷ |
| rs7748382 | 6 | 20665549 | A | G | 0.4008 | 0.2077 | 0.0249 | 8.27 × 10⁻¹⁷ |
| rs6456368 | 6 | 20659806 | T | C | 0.5992 | -0.2078 | 0.0250 | 9.30 × 10⁻¹⁷ |
| rs9295474 | 6 | 20652717 | C | G | 0.5923 | -0.2069 | 0.0249 | 9.33 × 10⁻¹⁷ |

Abbreviations: SNP, single nucleotide polymorphism; CHR, chromosome; POS, base-pair position; EFF_ALLELE, effect allele; NONEFF_ALLELE, non-effect allele; EFF_ALLELE_FREQ, effect allele frequency; BETA, effect size; SEBETA, standard error of the effect size.
